# Inflammation-Related Signature Profile Expression as a Poor Prognosis Marker after Oxaliplatin Treatment in Colorectal Cancer

**DOI:** 10.3390/ijms24043821

**Published:** 2023-02-14

**Authors:** Toni Martinez-Bernabe, Jordi Oliver, Jorge Sastre-Serra, Daniel Gabriel Pons

**Affiliations:** 1 Grupo Multidisciplinar de Oncología Traslacional, Institut Universitari d’Investigació en Ciències de la Salut (IUNICS), Universitat de les Illes Balears, 07122 Palma de Mallorca, Spain; 2 Instituto de Investigación Sanitaria de las Islas Baleares (IdISBa), Hospital Universitario Son Espases, Edificio S, 07120 Palma de Mallorca, Spain; 3 Ciber Fisiopatología Obesidad y Nutrición (CB06/03), Instituto Salud Carlos III, 28029 Madrid, Spain

**Keywords:** inflammation, colorectal cancer, tumourspheres, oxaliplatin, signature profile

## Abstract

Oxaliplatin is successfully used to eradicate micro-metastasis and improve survival, whereas the benefit of adjuvant chemotherapy in the early stages of colorectal cancer remains controversial. Inflammation plays a crucial role in colorectal cancer tumorigenesis. Inflammatory mechanisms are mediated by different immune cells through different cytokines, chemokines, and other proinflammatory molecules that trigger cell progression, an increase of cancer stem cell population, hyperplasia, and metastasis. This study focuses on the analysis of the oxaliplatin effect on tumourspheres formation efficiency, cell viability, cancer stem cells and stemness marker mRNA expression, as well as inflammation-related signature profile expression and its prognosis in primary- and metastatic-derived colorectal tumourspheres derived from colorectal cell lines isolated from the same patient 1 year apart. The results indicate that primary-derived colorectal tumourspheres respond to oxaliplatin, adapting to the adverse conditions through the modulation of CSCs and the stemness properties of tumourspheres. However, metastatic-derived colorectal tumourspheres response led to the release of cytokines and chemokines, promoting an inflammatory process. In addition, the expression of inflammatory markers showing greater difference between primary and metastatic tumours after oxaliplatin treatment correlates with poor prognosis in KM survival studies and is associated with a metastatic phenotype. Our data demonstrated that oxaliplatin triggers an inflammation-related signature profile expression in primary-derived colorectal tumourspheres, related with poor prognosis and a metastatic phenotype, which allow the tumour cells to adapt to the adverse condition. These data highlight the need for of drug testing and personalized medicine in the early stages of colorectal cancer.

## 1. Introductions

Colorectal cancer (CRC) is the third leading cause of cancer-mortality rate globally and the third most diagnosed cancer [1]. In 2020, 900,000 CRC-related deaths were reported, with 1.9 million patients diagnosed [1]. After diagnosis, CRC is treated by surgical resection, radiotherapy, and a combination of chemotherapies [2]. One of the most common chemotherapies used after resection is oxaliplatin, which acts by blocking DNA synthesis and replication [2,3]. Oxaliplatin links to DNA, forming mono- and biadducts, which implies a highly cytotoxic function [4]. Among the platin drugs, some DNA lesions caused by oxaliplatin are specific, affecting specific mechanisms of DNA repair, resulting in irreversibility of the lesions [5]. The efficiency of oxaliplatin in improving overall survival and preventing micro-metastasis has been proven in the last years in advanced colorectal cancer [6]. Nonetheless, the benefits of this drug in early tumour stages are unclear [2,7]. Notwithstanding various clinical trials, there is a lack of knowledge about the reason why early stages of CRC lead to relapse after oxaliplatin treatment [8,9]. In fact, almost 40% of patients present a relapse or even develop late metastasis, worsening the prognosis [10]. CRC triggers four different tumour stages (I, II, III, and IV) that also could be classified by tumour, node, and metastasis (TNM) staging [11]. TNM staging classifies the tumour through different features, such as size of the tumour (T), the spreading to the lymph nodes (N), and spreading to a different organ (M) [11]. Along these stages, various processes play relevant roles, such as inflammation [12,13].

Inflammatory mechanisms are mediated by different immune cells through different cytokines, chemokines, and other proinflammatory molecules, which trigger cell progression, hyperplasia, and metastasis [14,15]. These inflammatory mechanisms are mediated by interleukins and their receptors, creating an interconnection between tumour and immune cells [15]. In fact, CRC tumour cells release not only chemokines, which can recruit essential immune cells for the inflammatory process, but also interleukin, which allow the tumour cells to avoid apoptosis, trigger metastasis, and increase the cancer stem cell (CSC) population [12,15,16]. Furthermore, inflammation plays a critical role in CSC self-renewal as well by cytokine signalling pathways triggered by interleukin-6 (IL6), CXCL8, and interleukin-20 (CCL20), among others [16]. Recently, the reasons behind this relapse and metastasis have been considered, highlighting the Cancer Stem Cell (CSC) population among them [17]. CSCs are undifferentiated tumour cells that acquire stemness properties, allowing them to avoid cancer treatment and trigger relapse and metastasis [18]. Recently, different CSC markers have been revealed that distinguish CSCs from tumour cells [19]. However, each type of cancer presents specific CSCs and stemness markers on their CSCs, such as SOX2, OCT4, and ALDH [18,19]. Various authors have described the main CSCs markers of CRC, such as LGR5, CD133, and CD44, by identifying them from CRC tumours and testing the different subpopulations that exist within the tumour [20]. In addition, the effect of different drugs on CSC marker expression has not been elucidated, though it is known that specific conditions could decrease those markers [16,18]. These pathways promote the tumour microenvironment, allowing tumour cells to migrate and escape from the original tumour, but also maintaining metastatic mesenchymal and stem-like phenotypes [19]. 

Thus, our aim was to study the different effect of oxaliplatin on inflammation and CSC markers in primary and metastatic colorectal tumourspheres to determine why primary tumours recur or metastasize after chemotherapy treatment, while patients with metastatic tumours respond efficiently to this drug. To achieve this point, the present work focuses on the effect of oxaliplatin on cell viability, cell cycle, sphere formation efficiency and size, inflammatory markers, CSCs, and stemness in primary- and metastatic-derived colorectal cell line tumourspheres. It also investigates the correlation of the data obtained in tissue-specific TNMplots as well as gene expression- and signature profile expression-related overall survival and disease-free survival curves.

## 2. Results

### 2.1. Oxaliplatin Has a Different Effect on Cell Viability of SW480 and SW620 Colonospheres

To evaluate the oxaliplatin effect on tumour colonospheres, cell viability and cell cycle of SW480 and SW620 tumourspheres, primary- and metastatic-derived colorectal cell lines taken out from the same patient, 1 year apart, were analysed. As seen in Table 1, SW620 tumoursphere viability was decreased after oxaliplatin treatment, with a strong statistically significant reduction (36%: *p* < 0.001), while SW480 tumourspheres presented a slight decrease (around 10%: *p* < 0.001).

**Figure 1:**
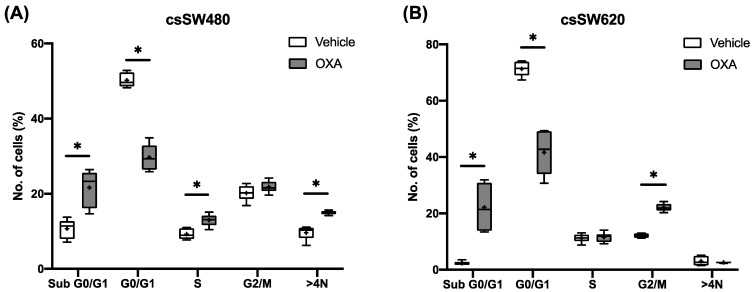
Cell cycle represented by number of cells (%) for SW480 (**A**) and SW620 (**B**) tumourspheres after 96 h of vehicle (0.1% DMSO) or oxaliplatin 5 µM treatment. Each box represents the interquartile range (25th to 75th percentiles), the central horizontal line is the median value, and the whiskers represent the minimum and the maximum values (*n* ≥ 4). Student’s test or Mann–Whitney test were performed to determine the significance between the experimental groups. Statistical significance was set at * *p* < 0.05.

To further analyse the effect of oxaliplatin treatment on cellular growth, cell cycle was analysed. SW480 tumourspheres showed an increase of sub G0/G1 (*p* = 0.001), S (*p* < 0.001) and >4 N (*p* = 0.009) phases in an oxaliplatin-treated condition, while SW620 tumourspheres increased both sub G0/G1 (*p* < 0.001) and G2/M (*p* < 0.001) phases. Moreover, oxaliplatin decreased G0/G1 phases in both SW480 (*p* < 0.001) and SW620 (*p* = 0.002) tumourspheres (Figure 1A,B).

### 2.2. Sphere Formation Was Modulated through Regulation of CSCs and Stemness Marker Expression after Oxaliplatin Treatment

**Figure 2:**
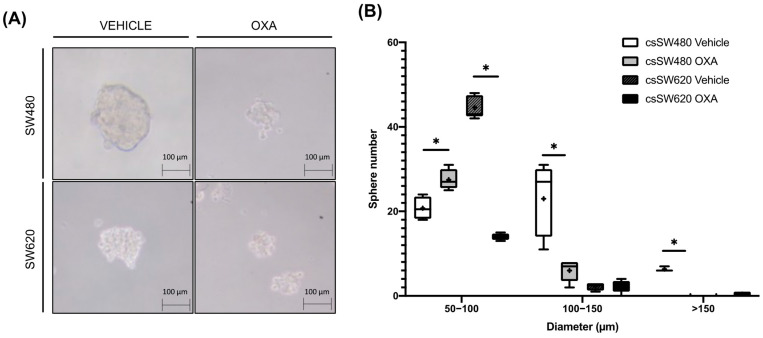
(**A**,**B**) Representative images of SW480 and SW620 tumourspheres after 96 h of vehicle (0.1% DMSO) or oxaliplatin 5 µM treatment for SW480 and SW620 tumourspheres after 96 h of oxaliplatin treatment. Each box represents the interquartile range (25th to 75th percentiles), the central horizontal line is the median value, and the whiskers represent the minimum and the maximum values (*n* ≥ 4). Student’s test or Mann–Whitney test were performed to determine the significance between the experimental groups. Statistical significance was set at * *p* < 0.05.

To analyse the sphere formation capacity of both SW480 and SW620 cells after oxaliplatin treatment, sphere formation efficiency (SFE) assay was performed. As seen in Table 2, SW620 tumourspheres showed a statistically significant decrease (*p* < 0.001) in SFE after oxaliplatin treatment, unlike SW480 tumourspheres (*p* = 0.11). However, as seen in representative images of Figure 2A,B, in addition, almost all SW620 tumourspheres presented a diameter of 50–100 µm, which was decreased (*p* < 0.001) after oxaliplatin treatment. Most SW480 tumourspheres presented a diameter of 50–100 and 100–150 µm, but also >150 µm. Even so, oxaliplatin showed a statistically significant decrease of both 100–150 µM (*p* = 0.009) and >150 µm (*p* = 0.003) SW480 tumourspheres, but an increase (*p* = 0.011) of 50–100 µm SW480 tumourspheres.

**Figure 3:**
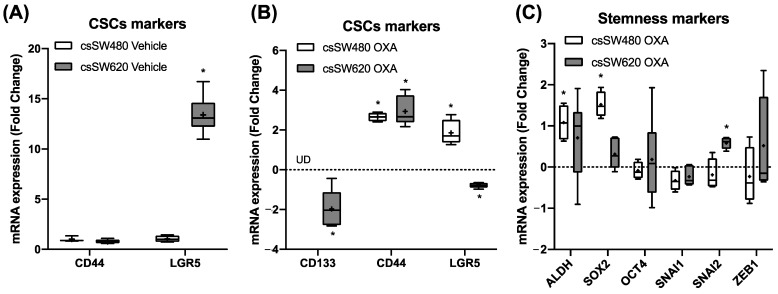
Cancer Stem Cell (CSC) marker mRNA expression after 96 h of vehicle (0.1% DMSO) (**A**) or oxaliplatin 5 µM (**B**) treatment in SW480 and SW620 tumourspheres. Stemness marker mRNA expression after 96 h of oxaliplatin treatment (**C**) in SW480 and SW620 tumourspheres. Each box represents the interquartile range (25th to 75th percentiles), the central horizontal line is the median value, and the whiskers represent the minimum and the maximum values (*n* ≥ 4). Student’s test or Mann–Whitney test were performed to determine the significance between the experimental groups. Statistical significance was set at * *p* < 0.05. UD: Undetected.

To further evaluate the presence of CRC-CSCs in the tumourspheres and their stemness after oxaliplatin treatment, mRNA expression of the main CRC-CSC and stemness markers were analysed. As seen in Figure 3A, SW620 tumourspheres presented higher levels of LGR5 (*p* < 0.001) mRNA expression than SW480 tumourspheres in a vehicle-treated condition. In addition, SW620 tumourspheres presented a statistically significant decrease of CD133 (*p* = 0.002) and LGR5 (*p* < 0.001) CSC markers mRNA expression, and an increase of CD44 (*p* < 0.001) expression after oxaliplatin treatment. In contrast, SW480 tumourspheres showed a statistically significant increase of LGR5 (*p* = 0.003) and CD44 (*p* < 0.001) mRNA expression, but CD133 mRNA expression was not detected after oxaliplatin treatment, as seen in Figure 3B. Moreover, ALDH (*p* = 0.024) and SOX2 (*p* = 0.038) mRNA expression were increased in SW480 after oxaliplatin treatment, while SW620 tumourspheres showed an increase of SNAI2 (*p* = 0.007) mRNA expression in an oxaliplatin-treated condition.

### 2.3. Bioinformatic Analysis Reveals Similar Inflammatory Poor Prognosis–Related Signature Profile Expression between Colorectal Cancer Metastases and Oxaliplatin-Treated SW480 Tumourspheres

**Figure 4:**
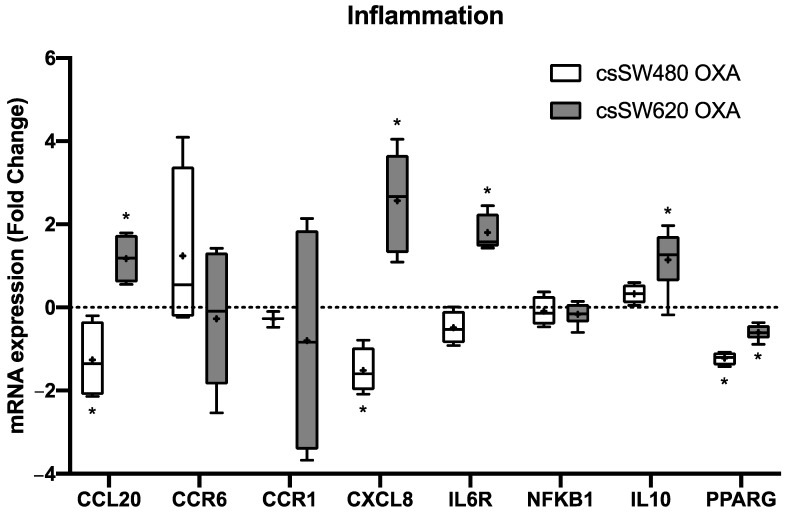
Inflammation marker mRNA expression after 96 h of oxaliplatin 5 µM treatment in SW480 and SW620 tumourspheres. Each box represents the interquartile range (25th to 75th percentiles), the central horizontal line is the median value, and the whiskers represent the minimum and the maximum values (*n* ≥ 4). Student’s test or Mann–Whitney test were performed to determine the significance between the experimental groups. Statistical significance was set at * *p* < 0.05.

To further investigate the inflammation condition of colon cancer cell tumourspheres after oxaliplatin treatment, mRNA expression of inflammation markers was analysed. As seen in Figure 4, SW480 tumourspheres showed a statistically significant decrease of inflammation-related marker mRNA expression CCL20 (*p* = 0.032), CXCL8 (IL8) (*p* = 0.006) and PPARG (*p* = 0.001). In contrast, SW620 tumourspheres presented an increase of CCL20 (*p* = 0.028), CXCL8 (*p* = 0.002), IL6R (*p* = 0.002) and IL10 (*p* = 0.009) inflammation-related marker mRNA expression, but a decrease in PPARG (*p* = 0.001) mRNA expression.

**Figure 5:**
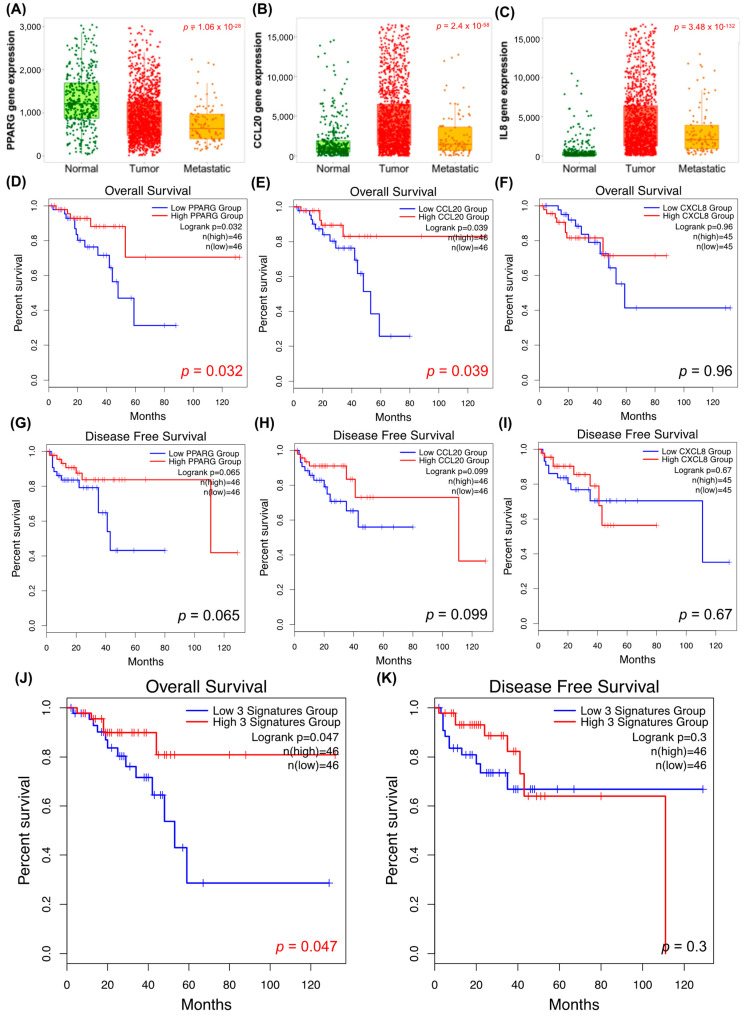
Analysis of PPARG (**A**), CCL20 (**B**) and CXCL8 (**C**) gene expression in normal, tumour and metastatic colon tissue. Overall survival and disease-free survival analysis of colorectal cancer patients based on GEPIA data. Kaplan–Meier survival analysis comparing high and low mRNA expression of PPARG (**D**), CCL20 (**E**), CXCL8 (**F**) and these three combined (**J**) regarding their associations with overall survival. Kaplan–Meier survival analysis comparing high and low mRNA expression of PPARG (**G**), CCL20 (**H**), CXCL8 (**I**) and these three combined (**K**) regarding their associations with disease-free survival.

To assess the relevance of the markers with differential expression, such as PPARG, CCL20 and CXCL8 (IL8), each gene’s expression level in normal, tumour and metastatic colon tissue was analysed by TNMplot. The median PPARG gene expression in normal tissue was 1230 (Q1: 882, Q3: 1752) compared to 807.5 (Q1: 481, Q3:1271) in tumour tissue and 635 (Q1: 397.5, Q3: 970) in metastatic tissue (*p* = 1.06 × 10^−28^, Kruskal–Wallis test; Figure 5A). PPARG expression was significantly decreased in tumours compared to normal but increased compared to metastatic colon tissue (post hoc Dunn test, *p* = 4.35 × 10^−26^ and *p* = 1.14 × 10^−16^, respectively). Moreover, the median CCL20 gene expression in normal tissue was 917 (Q1: 487, Q3: 1898) compared to 3339.5 (Q1: 1448, Q3: 7536) in tumour tissue and 1460 (Q1: 691, Q3: 3620.5) in metastatic tissue (*p* = 2.4 × 10^−58^, Kruskal–Wallis test; Figure 5B). CCL20 expression was significantly increased in tumours compared to normal and metastatic colon tissue (post hoc Dunn test, *p* = 3.16 × 10^−56^ and *p* = 4.39 × 10^−3^, respectively). Additionally, the median CXCL8 (IL8) gene expression in normal tissue was 177 (Q1: 70, Q3: 469) compared to 3407 (Q1: 1399, Q3: 7577) in tumour tissue and 2070 (Q1: 910.5, Q3: 3912.5) in metastatic tissue (*p*= 3.48 × 10^−132^, Kruskal–Wallis test; Figure 5C). CXCL8 expression was significantly increased in tumours compared to normal and metastatic colon tissue (post hoc Dunn test, *p* = 6.34 × 10^−134^ and *p* = 6.43 × 10^−22^, respectively).

In addition, to evaluate the prognosis of patients expressing PPARG, CCL20, CXCL8 and these three combined, overall survival and disease-free survival were analysed by the Kaplan—Meier method based on the log-rank test in GEPIA. Low mRNA expression of PPARG (*p* = 0.032) and CCL20 (*p* = 0.039) was associated with poor prognosis of CRC patients in terms of overall survival, as seen in Figure 5D,E. On the other hand, low signature profile mRNA expression based on PPARG, CCL20 and CXCL8 genes was significantly associated with poor prognosis of CRC patients in overall survival, as seen in Figure 5J.

## 3. Discussion

This study shows primary-derived colorectal tumoursphere response to oxaliplatin, adapting to the adverse condition modulating CSCs and stemness properties of tumourspheres, while metastatic-derived colorectal tumourspheres responded leading to release of cytokines and chemokines and promoting an inflammatory process. After early-stage CRC diagnosis and tumour resection, oxaliplatin is used as adjuvant chemotherapy to avoid relapse and completely eliminate tumours [2]. While oxaliplatin is successfully used to eradicate micro-metastasis and improve survival, the benefit of adjuvant chemotherapy in early stages of CRC remains controversial [2,7]. 

Over the last decades, inflammation has been described as a tumour progression factor and a hallmark of cancer [12,21]. Once the resection is done, an inflammatory process begins, implying a modulation of tissue structure and signalling molecule release [22]. Inflammation contributes to the emergence and development of cancer through the action of different immune cells, but also by the release of a variety of growth factors, cytokines, and chemokines [13,16,23], as we observed in our tumourspheres. We observed an increase of inflammatory-related protein (CCL20, IL-8, IL-6RA, IL-10, PPARG) mRNA expression in metastatic-derived tumourspheres after oxaliplatin treatment, which may be due to a loss of cell viability and tumoursphere efficiency formation. These inflammatory-related proteins play relevant roles in cancer development, such as IL-6R, IL-8 and IL-8RA, which induce tumoral progression, myeloid-derived suppressor cells (MDSCs) chemoattracting, angiogenesis activation, and stem cell property regulation [24,25,26]. We observed that metastatic-derived tumourspheres, which remain after oxaliplatin treatment, are more proliferative, as was revealed in the cell cycle analysis. Moreover, primary-derived tumourspheres showed a decrease of some inflammatory-related marker (CCL20, IL-8, and PPARG) mRNA expression after oxaliplatin treatment, most likely due to a slight decrease of cell viability. This decrease of CCL20, IL-8 and PPARG gene expression and the high proliferation status of primary colorectal tumourspheres after oxaliplatin treatment strongly correlate with a metastatic phenotype, as we observed in our TNMplot data for normal, tumorous, and metastatic tissues. Additionally, low mRNA expression of the CCL20 inflammation marker is highly correlated with poor prognosis, as we observed in Kaplan–Meier survival curves.

Furthermore, PPARG has been described as a colorectal tumour suppressor with anti-inflammatory and immunomodulatory capacities [27], in concordance with our TNMplot and Kaplan–Meier curve results. The data presented show the upregulation of inflammation marker mRNA expression in metastatic-derived colorectal tumourspheres after oxaliplatin treatment, except PPARG. Thus, primary-derived colorectal tumourspheres do not release proinflammatory chemokines and cytokines as much as metastatic-derived colorectal tumourspheres. This scenario could be explained as primary-derived colorectal tumourspheres efficiently responding to oxaliplatin treatment, maintaining tumoursphere formation but also increasing CSCs and stemness marker mRNA expression. It is worth noting that tumoural cells change the tissue architecture, which causes stress in the cells of stroma and induces the release of soluble inflammatory mediators and growth factors [12,15]. These factors maintain an inflammatory microenvironment, promoting tumorigenesis. In addition, the inflammatory process could be perpetuated by a positive feedback loop created by cytokines released by various immune cells [28]. According to our data, metastatic-derived colorectal tumourspheres lead to a release of cytokines, responding to a loss of cell viability and oxaliplatin-derived damage, to promote an inflammatory process that could be favourable for them.

In addition, primary-derived tumourspheres did not decrease sphere formation efficiency after oxaliplatin treatment as metastatic-derived tumourspheres did. Nonetheless, we found a decrease of primary-derived tumoursphere diameter in the oxaliplatin-treated condition, which reached >150 µm diameter in the vehicle-treated condition, except for metastatic-derived tumourspheres. Tumoursphere generation enriches the cell culture with cancer stem cells (CSCs), which present different features allowing them to resist cancer therapies [29]. CSCs express specific markers that allowed us to identify them, though these CSC markers are different among all cancer types [19,29]. CD44 and LGR5 colorectal CSC marker mRNA expression was detected in the primary- and metastatic-derived tumourspheres. However, LGR5 mRNA expression was decreased in metastatic-derived colorectal tumourspheres after oxaliplatin treatment, while it was increased in primary-derived colorectal tumourspheres. These data correlate with oxaliplatin’s different effect on tumoursphere formation efficiency and cell viability. LGR5 is tightly related to tumorigenesis, chemotherapy resistance and relapse in colorrectal cancer [30,31]. Additionally, LGR5 is highly expressed in the IV stage of colorectal cancer, being a poor prognosis marker and a potential target of colorectal cancer [32]. These data support the idea that oxaliplatin could induce a metastatic phenotype in primary colorectal cancer. In fact, primary and metastatic colorectal tumourspheres showed three differential mRNA expressions (PPARG, CCL20 and IL-8) in response to oxaliplatin treatment, which all were underexpressed in primary colorectal tumourspheres, differentially to metastatic colorectal tumourspheres, creating a signature profile. The data presented here show that low mRNA expression of the mentioned signature profile is strongly correlated with poor prognosis. Thus, primary-derived colorectal tumourspheres not only resisted oxaliplatin treatment, as we demonstrated in a previous study, but also could become more aggressive than before, increasing CSCs and stemness marker mRNA expression, leading to a poor prognosis. On the other hand, CD133 mRNA expression was only detected on metastatic-derived colorectal tumourspheres, as other authors have described [33]; however, was decreased after oxaliplatin treatment, in concordance with recent studies [34]. In fact, a decrease of CD133 mRNA expression has been related with treatment sensitivity [34].

In this way, the decrease of inflammation marker mRNA expression in primary-derived colorectal tumourspheres after oxaliplatin treatment could be caused by an acquisition of stemness and CSC properties, which would explain the response capacity to the oxaliplatin condition after 96 h. This condition would imply a low probability of survival for patients. The opposite occurs in metastatic-derived colorectal tumourspheres, which were highly affected by oxaliplatin treatment and responded with a release of both cytokines and chemokines to initiate an inflammatory process.

## 4. Materials and Methods

### 4.1. Reagents

Dulbecco’s modified Eagle’s medium High Glucose (DMEM) was purchased from BIOWEST (Riverside, MO, USA); Fetal Bovine Serum (FBS) and antibiotics (penicillin and streptomycin) from Biological Industries (Kibbutz Beit Haemek, Israel). 3D Tumoursphere Medium, its Supplementation Mix and DetachKit were purchased from PromoCell GmbH (Heidelberg, Germany). Oxaliplatin and Tri Reagent^®^ were purchased from Sigma-Aldrich (St. Louis, MO, USA). Routine chemicals were supplied by Bio-Rad Laboratories (Hercules, CA, USA), Sigma-Aldrich (St. Louis, MO, USA) and Panreac (Barcelona, Spain). The 6-well and 96-well ultra-low attachment (ULA) plates were purchased from SPL Life Sciences (Gyeonggi-do, Republic of Korea).

### 4.2. Colon Tumoursphere Generation

Colorectal cancer cell lines belonging from the same patient obtained 1 year apart, SW480 primary tumour-derived cells (CCL-228) and SW620 metastatic cancer-derived cells (CCL-227) were purchased from American Type Culture Collection ATCC (Manassas, VA, USA) within the last 3 years and maintained in DMEM supplemented with 10% FBS and 1% antibiotics (penicillin and streptomycin) at 37 °C with 5% CO_2_. For primary tumoursphere generation, 4 × 10^4^ cells/well were seeded in 6-well ULA plates with 3D Tumoursphere Medium supplemented with Supplementation Mix for 96 h. For subculture of tumourspheres, cells were dissociated enzymatically and mechanically using DetachKit and pipetting, respectively. The spheres were serially subcultured every 96 h until a third generation was obtained.

### 4.3. Sphere-Forming Efficiency (SFE) Assay

Secondary tumourspheres were dissociated, and 1.000 cells/well were seeded in 96-well ULA plates with vehicle or Oxaliplatin in 3D Tumoursphere Medium supplemented with Supplementation Mix for 96 h. Sphere-forming efficiency (SFE) was calculated as a percentage by dividing the tumourspheres formed per well by the number of cells seeded in the well. Images at 100× magnification (10× objective magnification and 10× ocular magnification) of each well were taken under a light inverted microscope. Diameter of tumourspheres was measured by ImageJ software 1.53a.

### 4.4. Cell Viability Assay

Cell viability was determined by fluorometric assay by staining DNA with Hoechst 33,342 from Sigma-Aldrich (St. Louis, MO, USA). Third generation tumourspheres were dissociated, and 1.5 × 10^4^ cells/well were seeded in 96-well plates with supplemented DMEM. After 24 h, cells were treated with vehicle (0.1% DMSO) or Oxaliplatin 5 µM in 3D Tumoursphere Medium supplemented with Supplementation Mix for 96 h. DNA was stained with 0.01 mg/mL Hoechst 33,342 in PBS. The plate was incubated for 5 min at 37 °C in 5% CO_2_, and fluorescence measurement was performed using an FLx800 microplate fluorescence reader (BIO-TEK, Winooski, VT, USA). Excitation and emission wavelengths were set at 360 nm and 460 nm, respectively.

### 4.5. Cell Cycle Assay

Cell cycle analysis was done by flow cytometry. SW480 and SW620 s generation tumoursphere cells were dissociated with DetachKit and seeded in 6-well ULA plates with vehicle or Oxaliplatin in 3D Tumoursphere Medium supplemented with Supplementation Mix for 96 h. Then, cells were detached and dissociated enzymatically and mechanically using DetachKit and pipetting, respectively, and fixed in cold 100% methanol. Fixed cells were incubated at −20 °C overnight and then centrifugated for 5 min at 600× *g*. Before the analysis, for DNA staining, cells were incubated at room temperature in the dark for 30 min with an RNAase and propidium iodide mix [35]. Flow cytometry experiments were performed using a Beckton-Dickinson FACSVerse flow cytometer, and the results were analysed with FACSuite v1.0.6 software.

### 4.6. RT-qPCR

Secondary tumoursphere cells were dissociated and seeded in 6-well ULA plates with vehicle or Oxaliplatin in 3D Tumoursphere Medium supplemented with Supplementation Mix for 96 h. RNA from cultured cells was isolated using Tri Reagent^®^ following the manufacturer’s protocol. Total RNA was quantified using a BioSpec-nano spectrophotometer (Shimadzu Biotech, Kyoto, Japan) set at 260 nm and 280 nm, obtaining a 260/280 nm ratio. cDNA was obtained by retrotranscription, and PCR reactions were carried out as previously reported [36]. Genes and their primers and annealing temperatures are shown in Supplementary Table S1. B2M and HMBS were used as housekeeping genes.

### 4.7. TNMplot

The gene expression analysis of PPARG, CCL20 and IL8 in normal, tumorous and metastatic tissue was performed in accordance with the website www.tnmplot.com (accessed on 24 September 2022). Details on the database are listed in the original publication [37]. Gene chip mRNA expression data extracted from the Gene Expression Omnibus of the National Center for Biotechnology Information (NCBI-GEO) for a sample collection of 377 normal colorectal tissues, 1450 colorectal cancer tissues, and 99 colorectal cancer metastases were analysed for PPARG, CCL20 and IL8 expression levels.

### 4.8. Overall Survival and Disease-Free Survival Analysis

Overall Survival (OS) and Disease-Free Survival (DFS) analysis of colorectal cancer patients was determined using GEPIA [38]. Cumulative event rate (OS) and time after treatment during which no sign of cancer is found (DFS) were determined by the Kaplan–Meier method, where the group cut-off was the median. PPARG, CCL20, IL8, and the signature profile expression of these were analysed.

### 4.9. Statistical Analysis

The statistical analyses were performed with the Statistical Program for the Social Sciences software for Windows (SPSS, version 27.0; SPSS Inc., Chicago, IL, USA). Data are presented as mean ± standard deviation (SD). The Shapiro–Wilk test was performed to check whether variables followed a normal distribution. The statistical differences between vehicle- and oxaliplatin-treated cells, and between vehicle conditions of both cell lines, were analysed using a Student’s *t*-test or Mann–Whitney test, with statistical significance set at *p* < 0.05 (*). TNMPlot data were analysed by the Kruskal–Wallis test using Dunn’s test for post hoc analysis in group comparisons. The null hypothesis that there is no difference between the control group and each of the test groups was rejected when the value of the minimal U statistic was smaller than the critical value. Survival curves stratified by risk factors were compared by log-rank test, considering *p* < 0.05 to indicate statistical significance.

## 5. Conclusions

Primary-derived colorectal tumourspheres adapted to the oxaliplatin condition, likely modulating CSCs and stemness properties of tumourspheres, while metastatic-derived colorectal tumourspheres responded, leading to the release of cytokines and chemokines and promoting an inflammatory process. Oxaliplatin enhanced a CSC-phenotype in primary-derived CRC tumourspheres, unlike what the outcome in metastatic-derived CRC tumourspheres. Thus, the data reinforce the idea of the efficiency of oxaliplatin in advanced stages of CRC but not in early stages. Further studies would be necessary to elucidate the adapting mechanisms to oxaliplatin treatment and provide an accurate signature profile expression of primary colorectal cancer to avoid relapses and improve overall survival. In addition, this study not only highlights the need to determine accurate drugs to target early stages of colorectal cancer, including for personalised medicine, but also shows a new signature profile expression that may be helpful for drug-testing.

## Figures and Tables

**Table 1:** Oxaliplatin effect on cell viability in SW480 and SW620 tumourspheres. CRC cell lines were treated 96 h with vehicle (0.1% DMSO) or 5 µM oxaliplatin. Data represent means ± SD (*n* ≥ 4). Student’s test or Mann—Whitney test were performed to find determine significance between the experimental groups. Statistical significance was set at * *p* < 0.05.

	csSW480		csSW620	
	Vehicle	OXA	Vehicle	OXA
Cell viability (%)	100 ± 3	90.3 ± 2.6 *	100 ± 2	64.0 ± 2.9 *

**Table 2:** Oxaliplatin effect on sphere formation efficiency in SW480 and SW620 tumourspheres. CRC cell lines were treated 96 h with vehicle (0.1% DMSO) or 5 µM oxaliplatin. Data represent means ± SD (*n* ≥ 4). Student’s test or Mann–Whitney test were performed to determine the significance between the experimental groups. Statistical significance was set at * *p* < 0.05.

	csSW480		csSW620	
	Vehicle	OXA	Vehicle	OXA
SFE (%)	4.8 ± 1.2	3.7 ± 0.5	4.8 ± 0.3	1.7 ± 0.3 *

## Data Availability

The data presented in this study are available on request from the corresponding author.

## Supplementary Materials

The following supporting information can be downloaded at: https://www.mdpi.com/article/10.3390/ijms24043821/s1.
