# Supplementary material for: Inflammation-Related Signature Profile Expression as a Poor Prognosis Marker after Oxaliplatin Treatment in Colorectal Cancer
Source: Int J Mol Sci. 2023 Feb 14;24(4):3821. doi: 10.3390/ijms24043821 (PMC9965239; doi:10.3390/ijms24043821)
Supplement: Supplementary file 1 [file ijms-24-03821-s001.zip › ijms-2155946-supplementary.pdf]

Table S1: Primers and conditions used for RT-qPCR

Gene Accession number	Forward Primer (5'-3') Reverse Primer (5'-3')
ALDH *NM_000689.5*	CGGGAAAAGCAATCTGAAGAGGG GATGCGGCTATACAACACTGGC
B2M *NM_004048*	TTTCATCCATCCGACATTGA CGGCAGGCATACTCATCTTT
CCL20 *NM_004591.3*	GCAAGCAACTTTGACTGCTG CAAGTCCAGTGAAGGCACAAA
CCR1 *NM_000634.3*	AGTTCTTGGCACGTCATCGT CCCCTGAAGACACCAGTTCC
CCR6 *NM_004367.6*	ACCGCAGATAACGACAATGC CATGAGCACGTTAAGTCCCG
CD133 *NM_006017.3*	CATGCTCTCAGCTCTCCCG ATAAACAGCAGCCCCAGGAC
CD44 *NM_000610.4*	CACACCCTCCCCTCATTCAC TGGATGGCTGGTATGAGCTG
CXCL8 *NM_000584.4*	GGCACAAACTTTCAGAGACAGCAG GTTTCTTCCTGGCTCTTGTCCTAG
HMBS *NM_001024382.2*	GAGAAGTCCAAGCAACAGC CCTTCAGAACTGGTTTATTAGTAGG
IL6R *NM_000565.4*	TGGGAGGTGGAGAAGAGAGA AGGACCTCAGGTGAGAAGCA
IL10 *NM_000572.3*	ACATCAAGGCGCATGTGAAC CACGGCCTTGCTCTTGTTTTC
NFKB1 *NM_021975.4*	GCCGGGATGGCTTCTATGAG CATTCAGGTCGTAGTCCCCAC
OCT4 *NM_203289.6*	CTTGAATCCCGAATGGAAAGGG GTGTATATCCCAGGGTGATCCTC
PPARG *NM_138712.5*	GAGCCCAAGTTTGAGTTTGC CTGTGAGGACTCAGGGTGGT
SNAI1 *NM_005985.4*	ACCCCAATCGGAAGCCTAAC TCCCAGATGAGCATTGGCAG
SNAI2 *NM_003068.5*	ACGCCTCCAAAAAGCCAAAC ACTCACTCGCCCCAAAGATG
SOX2 *NM_003106.4*	GCCGAGTGGAAACTTTTGTCG GGCAGCGTGTACTTATCCTTCT
ZEB1 *NM_001128128.3*	CTGCTGGGAGGATGACACAG ATGACCACTGGCTTCTGGTG
